# The Emerging Role of TLR and Innate Immunity in Cardiovascular Disease

**DOI:** 10.1155/2012/181394

**Published:** 2012-04-22

**Authors:** Rolf Spirig, Janice Tsui, Sidney Shaw

**Affiliations:** ^1^CSL Behring AG, 3000 Bern, Switzerland; ^2^Division of Surgery & Interventional Science, University College London, Royal Free Campus, London NW3 2QG, UK; ^3^Department of Clinical Experimental Research, University of Bern, 3010 Bern, Switzerland

## Abstract

Cardiovascular disease is a complex disorder involving multiple pathophysiological processes, several of which involve activation of toll-like receptors (TLRs) of the innate immune system. As sentinels of innate immunity TLRs are nonclonally germline-encoded molecular pattern recognition receptors that recognize exogenous as well as tissue-derived molecular dangers signals promoting inflammation. In addition to their expression in immune cells, TLRs are found in other tissues and cell types including cardiomyocytes, endothelial and vascular smooth muscle cells. TLRs are differentially regulated in various cell types by several cardiovascular risk factors such as hypercholesterolemia, hyperlipidemia, and hyperglycemia and may represent a key mechanism linking chronic inflammation, cardiovascular disease progression, and activation of the immune system. Modulation of TLR signaling by specific TLR agonists or antagonists, alone or in combination, may be a useful therapeutic approach to treat various cardiovascular inflammatory conditions such as atherosclerosis, peripheral arterial disease, secondary microvascular complications of diabetes, autoimmune disease, and ischemia reperfusion injury. In this paper we discuss recent developments and current evidence for the role of TLR in cardiovascular disease as well as the therapeutic potential of various compounds on inhibition of TLR-mediated inflammatory responses.

## 1. Introduction: Innate Immunity and Toll-Like Receptors (TLRs)

Historically the immune system has been divided into the innate and the adaptive immune system. Neutrophils, eosinophils, basophils, mast cells, monocytes, macrophages, dendritic cells (DCs), NK cells, NK-T cells, *γδ* T cells, and B-1 cells are considered to be cellular members of the innate immune system which can be activated by signaling through TLR. In addition, endothelial cells may form part of this system since they also possess antigen-presenting capabilities and therefore immune regulation properties apart from their function as a barrier between tissue and blood [1]. 

A year after the discovery of the role of drosophila Toll protein in the host defense against fungal infection [2], a mammalian homologue was identified, referred to as TLR4 [3]. Since then, 13 members of the TLR family have been identified in mammals, ten in humans, and twelve in mice. Mice do not express TLR10 but do express TLR11, TLR12, and TLR13 [4]. TLR1, TLR2, TLR4, TLR5, TLR6, and TLR11 are displayed on the cell surface while TLR3, TLR7, TLR8, and TLR9 are localized intracellularly. TLRs are distributed and differentially expressed in several cell types and tissues. They are present on polymorphonuclear cells, macrophages, mast cells, DC, NK cells, T cells, and B cells. Interestingly, TLR expression has also been detected on cardiac, epithelial, endothelial, and vascular smooth muscle cells. Moreover, mesenchymal and parenchymal cells of different organs and tissue such as kidney, heart, lung, liver, skin, brain and intestine express TLR, but their functional role and relevance is not yet fully understood [5].

The molecular weight of TLR ranges between 90 and 115 kDa. The extracellular region of Toll contains leucine-rich repeat (LRR) motifs whereas the cytoplasmic domain has similarities with that of the mammalian Interleukin-1 receptor (IL-1R) family and is designated as Toll/IL-1R (TIR) homology domain, containing around 200 amino acids. Within this domain, the regions of homology comprise three conserved boxes, which are crucial for signaling. After ligand binding, TLRs dimerize and undergo the conformational change required for recruitment of downstream signaling molecules. In general, these include the adaptor molecule, myeloid differentiation primary-response protein 88 (MyD88), TIR-domain-containing adaptor protein (TIRAP; also known as MyD88-adaptor-like protein or Mal), IL-1R-associated kinases (IRAKs), transforming growth factor-*β*- (TGF-*β*-) activated kinase (TAK1), TAK1-binding protein 1 (TAB1), TAB2, and tumor-necrosis factor (TNF) receptor-associated factor 6 (TRAF6) [6, 7]. The TLR family signaling pathway is highly homologous to that of the IL-1R family and represents the core pathway of all TLR, except for TLR3. Studies in 2001 revealed the existence of a MyD88-independent pathway since stimulation of MyD88-deficient DC with LPS still induced their maturation [8]. Therefore, exposure to LPS induces TLR4-signaling via a MyD88-dependent as well as MyD88-independent pathway, which subsequently activates IRF3 [9]. Up to now, TLR3-signaling is considered to be MyD88 independent [7]. Importantly, there seems to be a difference between TLR signaling induced by endogenous versus exogenous ligands which may in part be due to differential TLR signaling complex formation [10].

## 2. Toll-Like Receptors in Autoimmune and Cardiovascular Disease

**Figure 1:**
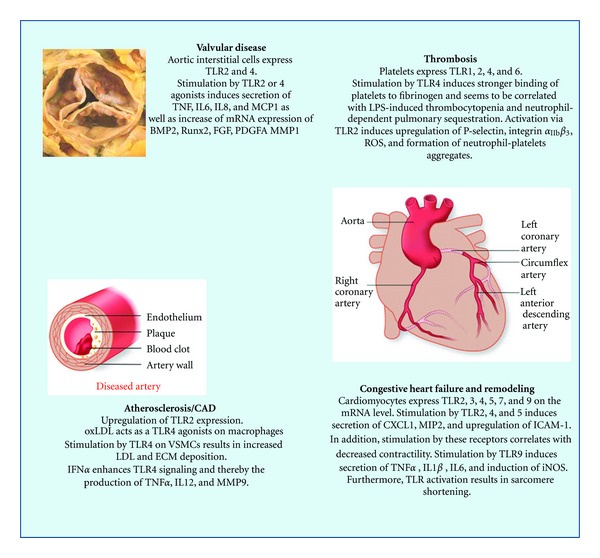
Summary of current data implicating TLR signaling in various cardiovascular disease processes (figure modified from [13]). BMP: bone morphogenetic protein; Runx2: Runt related transcription factor 2; FGF: fibroblast growth factor; PDGFA: platelet derived growth factor A; MMP: matrix metalloproteinase; ROS: reactive oxygen species; VSMC: vascular smooth muscle cells; ECM: extracellular matrix; LDL: low density lipoprotein; CXCL1: chemokine (C-X-C motif) ligand 1; MIP: macrophage-inflammatory protein; ICAM: intracellular adhesion molecule; iNOS: inducible nitric oxide synthase.

The concept that innate immune signaling can be triggered not only by external pathogens but also by endogenous molecules released in response to tissue injury was first proposed almost two decades ago by Matzinger [11]. Subsequently, it was shown that antigen-presenting cells, namely, DC, can be activated by a variety of endogenous stimuli [12]. Since then further advances in this area have been surprisingly slow and only recently their emerging potential role in cardiovascular disease has become to be recognized as summarized in Figure 1 [13]. 

In this context, several endogenously derived molecules released from necrotic cells, so-called damage-associated molecular patterns (DAMPs) or alarmins, have been identified, which lead to “sterile inflammation” via activation of TLR. In addition, other molecules released from dying cells, for example, proteinases also lead to the generation of extracellular DAMP by degradation of components of the extracellular matrix or glycocalyx. DAMPs now encompass a wide range of molecules including heat shock proteins, high-mobility group box 1 (HMGB1) protein, a chromatin-binding nuclear protein, ATP, uric acid, heparan sulfate (HS), hyaluronan, and others [14, 15]. These molecules have been shown to bind to different TLRs or other molecular pattern recognition receptors (PRRs) expressed on various cell types and trigger the release of proinflammatory mediators. 

### 2.1. Atherosclerosis: Interaction of Risk Factors and TLR

Excessive accumulation of lipids in macrophages resulting in foam cell formation is a hallmark of atherosclerosis. TLR4 has been shown to contribute to early-stage intimal foam cell accumulation at lesion-prone aortic sites in ApoE KO mice, as does TLR2 to a lesser extent [16]. Intimal smooth muscle cells surround and penetrate early lesions, where TLR4 signaling, enhanced by hypercholesterolemia, promotes lesion progression by stimulation of acyl-coenzyme A: cholesterol acyltransferase-1 mRNA expression, cytoplasmic cholesterol ester accumulation, and monocyte chemoattractant protein-1 (MCP-1) mRNA and protein expression in a TLR4-dependent manner. Other TLRs also appear to be involved. Lipid accumulation in macrophages is closely linked to the PAT family of proteins (named after perilipin, adipophilin, and TIP47 (tail-interacting protein of 47 kDa). TLR9-mediated signaling stimulates perilipin 3 expression and macrophage accumulation of lipids, especially triglycerides. Oligodeoxynucleotide (ODN) 1826, an agonist ligand of TLR9, significantly enhanced perilipin 3 expression in RAW264.7 cells via upregulation of IL-1*α* and IFN*β*, whilst chloroquine, a TLR9 inhibitor, virtually completely abolished ODN1826-induced perilipin 3 expression. Inhibitors of c-jun NH2-terminal kinase and PI3-kinase suppressed the level of perilipin 3 mRNA induced by ODN1826 [17].

Not all effects of TLR activation, however, may be detrimental. Soluble forms of human TLR2 (sTLR2) have been shown to be released by monocytes, and depletion of sTLR2 resulted in an exaggerated inflammatory response [18]. Patients with postmyocardial infarction and heart failure have also been shown to have markedly decreased sTLR2 compared to controls [19].

Recent studies indicate that neointima formation in a perivascular collar-induced injury model is reduced by systemic administration of the dsRNA analog poly(I:C) (a TLR3 agonist) in a TLR3-dependent manner. Furthermore, genetic deletion of TLR3 markedly enhanced the development of elastic lamina damage after collar-induced injury and accelerated the onset of atherosclerosis in hypercholesterolemic ApoE knockout mice [20]. Collectively, these data suggest a protective role for TLR3 signaling in the vessel wall. Taken together, current data indicates that the contribution of TLR signaling to the progression of the atherosclerotic process may depend, at least in part, on a balance between detrimental and protective TLR-mediated mechanisms. This in turn may depend not only on changes in the relative expression of appropriate receptors on relevant cell types but also on the relative availability of endogenous ligands.

### 2.2. Diabetes, Insulin Resistance, and Other Cardiovascular Risk Factor Interactions with TLR

Other cardiovascular risk factors, notably diabetes, obesity, and insulin resistance, are also associated with a low-grade inflammatory state that reflects activation of innate immunity associated with metabolic, environmental, and genetic factors. Evidence suggests, for example, that resistin, originally described as an adipose tissue-specific hormone, is involved in pathologic processes leading to CVD including inflammation, endothelial dysfunction, thrombosis, angiogenesis, and smooth muscle cell dysfunction. Recent data indicates that a key mechanism underlying its detrimental effects in these processes is that TLR4 serves as a receptor for the proinflammatory effects of resistin in human cells [21, 22]. This may partly explain the multifunctional role of resistin in chronic inflammation, atherosclerosis, and insulin resistance. Similarly, nutritional fatty acids, whose circulating levels are often increased in obesity, activate TLR4 signaling in adipocytes and macrophages and the capacity of dietary fatty acids to induce inflammatory signaling in adipose cells or tissue and macrophages is blunted in the absence of TLR4.

Other studies suggest an association between the Asp299Gly polymorphism of the TLR4 gene and early onset of diabetic retinopathy in type 2 diabetic patients [23]. TLR2 and TLR4 expressions as well as signaling have also been shown to be enhanced in monocytes of patients with Type 1 diabetes with microvascular complications [24]. This may contribute to the accentuated proinflammatory state and complications of T1DM. Underlying molecular mechanisms linking these observations appear to involve complex-formation between advanced glycation end product-modified oxidized low-density lipoprotein (AGE-LDL), the receptor for advanced glycation end products (RAGE), and the scavenger receptor CD36 [25]. Subsequent activation of downstream signaling pathways induced by binding of this complex to TLR4 results in activation of p38-*α*, JNK, and ERK1 kinases and AP1, Elk1, and NF*κ*B transcription factors leading to increased production of TNF*α* and proinflammatory cytokines. These mechanisms may partly underlie the increased risk of atherosclerosis observed in diabetics. Two common polymorphisms in TLR4, D299G and T399I, were shown *in vitro* to reduce the response of TLR4 to LPS but had no effect on the AGE-LDL-complex signaling. This supports data from other studies suggesting that TLR activation by DAMP may activate alternative downstream proinflammatory pathways to those induced by pathogen-associated ligands.

## 3. Toll-Like Receptors in Cardiac I/R Injury

### 3.1. Toll-Like Receptors as Sentinels of Innate Immunity in Cardiac I/R Injury

**Figure 2:**
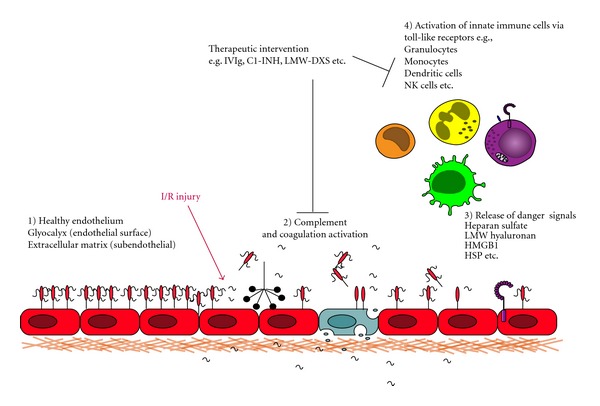
Cardiac I/R injury activates multiple inflammatory pathways such as complement, coagulation, and/or innate immune cells by binding of danger signals via expressed TLR. LMW-DXS: low molecular weight dextran sulfate, HMGB1: high-mobility group protein box 1, HSP: heat shock proteins.

There is an increasing number of studies demonstrating a major role of TLR in several animal models of ischemia reperfusion (I/R) injury. Cardiac I/R injury has a significant clinical relevance as, for example, in heart transplantation (HTx), myocardial infarction (MI), or coronary artery bypass graft surgery. Tissue damage and inflammation occurs after coronary artery occlusion (ischemia) when reperfusion occurs (restoration of blood flow). A hallmark of I/R injury is a strong activation of the innate immune system, that is, activation of complement and coagulation, recruitment of innate immune cells, cytokine release, formation of reactive oxygen species (ROS), mitochrondrial dysfunction, as well as apoptosis and cell necrosis (Figure 2). Studies with TLR deficient mice have demonstrated a crucial role of TLR2 and TLR4 in I/R injury-mediated inflammatory responses in the heart [26, 27]. Kaczorowski et al. showed in a murine cardiac transplantation model that serum levels of TNF-*α*, IL-1*β*, IL-6, troponin I, and MCP-1 were dramatically reduced in mice deficient in TLR4 signaling. Furthermore, these mice had reduced intragraft mRNA levels of  TNF-*α*, IL-1*β*, IL-6, EGR-1, ICAM-1, and iNOS [26]. In a mouse model of myocardial infarction, TLR4 deficiency resulted in less tissue damage and cardioprotection [28]. A cardioprotective effect has also been observed for mice deficient for TLR2, for example, by a reduced infiltration of neutrophils into the tissue [27]. The exact role of TLR2 however is somewhat controversial since, in other studies, TLR2 agonist ligands were reported to induce cardioprotection, mediated via a TLR2/PI3K/Akt-dependent mechanism [29]. The reason for these conflicting reports is currently unclear but may reflect differences between chronic and acute models of I/R injury.

### 3.2. Interplay of TLR with Other Members of Innate Immunity in Cardiac I/R Injury

I/R injury leads to the activation of multiple inflammatory pathways. Furthermore, there is an active interplay between pathways such as TLR and complement. The release of the nonmuscle myosin from dying cells is recognized by naturally occurring IgM antibodies, resulting in complement activation and tissue damage [30]. Interestingly, the anaphylatoxin and complement cleavage product C5a has been shown to negatively regulate production of IL-12 family members such as IL-12, IL-23, and IL-27 in inflammatory macrophages [31]. Furthermore, mice deficient in the membrane complement regulator CD55 (DAF) have elevated levels of TNF-*α*, IL-1*β*, and IL-6 in response to TLR agonists, whereas the levels of IL-12p40 are slightly decreased [32].

Interestingly, the endogenous TLR4 ligand heparan sulfate (HS) has been shown to induce the production of complement proteins C1q or C3 by human monocyte-derived DC (MoDC) [33] whereas exogenous LPS reduced secretion of C1q [34]. In addition, exogenous C1q seems to potentiate the LPS-induced secretion of IL-12p70 by human MoDC *in vitro * [35]. Another study has reported decreased levels of IL-12p70 secretion by DC after LPS-challenge in patients deficient for C1q [36].

Another important characteristic of I/R injury is the very low oxygen level during ischemia. Hypoxia induces the expression of the transcription factor hypoxia-inducible factor-1*α* (HIF-1*α*) which has been described as a key regulator of a broad range of cellular and systemic responses to hypoxic conditions. Recent studies suggest a cross-talk between HIF-1*α* and TLR. It has been shown that stimulation of human MoDC with LPS under hypoxic conditions resulted in a significantly higher expression of the costimulatory molecules CD80 and CD86 [37]. The same synergistic effect was observed for murine bone-marrow-derived DC [38]. In addition, HIF-1*α* regulates the expression of VEGF and endothelin-1 (ET-1), a potent vasoactive peptide known to be involved in cardiac I/R injury [39]. Stimulation of macrophages as well as MoDC with TLR agonists induces the secretion of VEGF and ET-1 [40, 41].

### 3.3. Strategies to Inhibit TLR Activation in Cardiac I/R Injury

Specific inhibition of TLR2 with a monoclonal antibody has been demonstrated to attenuate myocardial I/R injury in mice [42]. Similarly, eritoran, a synthetic lipid-A analogue which inhibits TLR4 signaling, was beneficial in a mouse model of myocardial infarction [43]. It should be noted however that I/R injury is a multifactorial injury involving many different inflammatory pathways and to target one single pathway might not be sufficient. Hence, combination therapies may show greater efficacy.

To date several compounds have been described, many of them already routinely used in the clinics for other indications, which have been shown to inhibit TLR signaling *in vitro* and to possess therapeutic potential in animal models of MI or HTx. Therefore, we will discuss in the following paragraphs compounds which have shown to have anti-inflammatory activity on various pathways of cardiac I/R injury such as the coagulation or complement cascade, TLR signaling, leukocyte recruitment, NK cell activation, maturation of DC, and others (summarized in Table 1).

#### 3.3.1. Low Molecular Weight Dextran Sulfate (LMW-DXS)

Low molecular weight dextran sulfate (LMW-DXS, MW: 5000 Dalton) has various anti-inflammatory properties. Inhibition of all three activation pathways of complement is mediated by binding to factor H [44] and enhancement of the activity of C1-INH [45]. Furthermore, LMW-DXS inhibits coagulation by enhancing the anticoagulatory activity of antithrombin III (ATIII) and C1-INH against activated factor XI [46]. Interestingly, LMW-DXS interferes with platelet adhesion [47] and has beneficial effects in different animal models of cardiac I/R injury. In a pig model of acute myocardial I/R injury, LMW-DXS significantly reduced infarct size [48] and facilitated anti-CD4 mAb-induced long-term cardiac allograft survival in rats despite prolonged cold graft ischemia [49]. LMW-DXS also inhibited TLR2- and TLR4-mediated maturation of human MoDC and prevented the upregulation of costimulatory molecules including CD40, CD80, and CD86 on MoDC in a dose-dependent manner. Secretion of the proinflammatory cytokines TNF-*α*, IL-6, and IL-1*β* was also significantly reduced. As a functional consequence of these effects, antigen-presentation to T cells was prevented. TLR-induced signal transduction, phosphorylation of I*κ*B-*α*, as well as activation of the downstream proinflammatory transcription factor NF*κ*B were also inhibited by LMW-DXS [33]. An associated *in vitro* study showed similar effects of LMW-DXS on TLR2-mediated activation of human NK cells. Phenotypic activation was significantly inhibited by LMW-DXS as shown by a reduced upregulation of CD25, CD56, CD69, as well as NKp44. Furthermore, release of IFN*γ* was significantly reduced and degranulation of NK cells was attenuated as evaluated by the upregulation of CD107a [50].  LMW-DXS also interferes with activation of MoDC by the endogenous TLR4 ligand, heparan sulfate proteoglycan (HSPG). This is rapidly released from the vascular endothelial surface under conditions of inflammation and tissue damage [51–53] by proteolytic cleavage of the protein core or by endoglycolytic cleavage of the HS chains [53, 54]. In the plasma of vascular surgery patients, elevated levels of syndecan-1 and HS were found as early as 15 minutes after reperfusion [55]. Free HS has been considered to act as DAMP, since it induces maturation of macrophages and DC via TLR4 [56–58]. 

Overall, LMW-DXS is well tolerated in humans and induces an increase in the anti-inflammatory hepatocyte growth factor (HGF) in plasma [59]. In addition it not only acts as a complement and coagulation inhibitor but also modulates innate immune cells as, for example, shown by inhibition of NK cell activation. Moreover, the crosstalk between innate and adaptive immunity is prevented by inhibition of TLR-mediated maturation of DC. Hence, LMW-DXS and similarly acting, nontoxic inhibitors of complement, coagulation, and TLR activation may be of therapeutic interest for the prevention of cardiac I/R injury.

#### 3.3.2. Intravenous Immunoglobulins (IVIgs)

IVIg products are derived from pooled human plasma of thousands of donors and have been used for decades as replacement therapy for patients with hypogammaglobulinemia, such as X-linked agammaglobulinemias or common variable immunodeficiencies. In addition, anti-inflammatory therapy with high-doses of IVIg is used clinically in a variety of acute or chronic autoimmune disease as, for example, Idiopathic Thrombocytopenic Purpura (ITP), Kawasaki Disease, or Guillain Barré Syndrome. Several anti-inflammatory properties of IVIg have been described for IVIg preparations including complement inhibition, anticytokine antibodies, inhibition of leukocyte rolling, induction of T regulatory cells, and others as published and reviewed by others [60–62]. Importantly, IVIg has successfully been used in the clinic as a combination therapy for a patient undergoing cardiac transplantation [63]. A few reports have demonstrated an inhibitory effect of IVIg on TLR-mediated activation of immune cells *in vitro*. One study investigated the effect of IVIg on differentiation of monocytes into MoDC as well as the effect of IVIg on TLR4-mediated maturation of MoDC. Pretreatment of MoDC with IVIg followed by stimulation with LPS resulted in reduced upregulation of the costimulatory molecules CD40, CD80, and CD86. Furthermore, upregulation of MHC class II, the key molecule for antigen-presentation, was prevented by IVIg. Interestingly, LPS- induced secretion of IL-12p70 was inhibited whereas the anti-inflammatory cytokine IL-10 was significantly upregulated. No inhibition was observed for the secretion of the proinflammatory cytokine TNF-*α*. Importantly, DC-mediated T cell proliferation analyzed in mixed leukocyte reaction (MLR) with allogeneic T cells was reduced by IVIg [64]. A recent study has further investigated the effect of IVIg on the differentiation of CD14 positive human monocytes into MoDC. Ballow and Allen demonstrated that IVIg primarily modulates the differentiation of “TLR-primed” monocytes before subsequent differentiation into MoDC by IL-4 and GM-CSF. Interestingly, priming with poly I/C (TLR3 agonist) in combination with IVIg did not result in an increase of CD83 expression, as observed for the other used TLR agonists [65]. Controversial results were observed regarding DC-induced proliferation of allogeneic T-cell. This study demonstrated an increase of T cell proliferation by IVIg-differentiated DC [65] whereas Bayry et al. have shown a decrease of T cell proliferation [64]. 

The effect of TLR9 stimulation by CpG oligonucleotides on B cells of SLE patients as well as healthy donors has been investigated. Coincubation of B cells with IVIg (10 mg/ml) and CpG oligos resulted in a decreased secretion of IL-6 as well as IL-10. IVIg had a similar inhibitory effect on the activation of human B cells isolated from SLE patients and healthy controls [66]. 

It would be of interest if IVIg also interferes with cell activation induced by DAMP. As shown by Bayry et al., IVIg preparations contain specific LPS antibodies [64]. Therefore, some of the observed inhibitory effects might be explained by binding and neutralization of the exogenous agonist used, namely LPS.

#### 3.3.3. C1-Esterase Inhibitor (C1-INH)

C1-INH has been reported to have various anti-inflammatory properties. Initially C1-INH has been characterized as a potent inhibitor of the classical pathway of complement activation. Follow-up studies provided evidence for inhibition of the mannan-binding lectin as well as the alternative pathway of complement activation. Protease inhibition has been reported for kallikrein, factor XI, factor XII, plasmin, tissue plasminogen activator (tPA), and thrombin. Furthermore, binding to neutrophils, macrophages, and endothelial cells (ECs) has also been shown [67]. In the clinic, C1-INH is commonly used to treat patients with C1-INH deficiencies who suffer from Hereditary Angioedemia (HAE). 

*In vivo*, treatment with C1-INH has been demonstrated to be beneficial in different models of cardiac I/R injury [68, 69]. In addition, C1-INH has successfully been used as a combined therapy for a patient undergoing cardiac transplantation [63]. Only one study has investigated the effect of C1-INH on TLR4- mediated activation of a murine macrophage cell line. Inhibition of LPS-mediated activation of murine macrophages seems to be mediated by binding and neutralization of LPS by C1-INH [70]. *N-*glycosylation of the protein seems to be important for binding of LPS [71]. Interestingly, C1-INH also binds to graft EC, while still maintaining its function as complement inhibitor as shown in a model of *ex vivo* perfused porcine livers [72] and could therefore potentially be used as an additive to organ preservation solutions in order to protect the graft from I/R injury.

#### 3.3.4. Antithrombin III (ATIII)

ATIII is a major inhibitor of the coagulation system and acts as a potent inactivator of thrombin and factor Xa. Clinically, ATIII concentrates are used to treat inherited ATIII deficiencies [73]. Apart from coagulation inhibition, ATIII has been shown to interfere with leukocyte adhesion *in vitro* and *in vivo* [74]. ATIII also possesses effective anti-inflammatory properties. Mansell et al. have demonstrated an inhibitory effect of ATIII on TLR4-mediated nuclear translocation of the proinflammatory transcription factor NF*κ*B in the human monocytic cell line THP1 [75]. There are no other reports so far showing an inhibitory effect of ATIII on other TLR agonist-induced activation of immune cells. Interestingly, in a mouse model of cardiac transplantation, high doses of ATIII have been shown to induce long-term graft survival. Induction of regulatory cells by ATIII has been suggested but not demonstrated by the authors [76].

#### 3.3.5. Alpha-1 Antitrypsin (*α*1AT)

*α*1AT is routinely used in the clinic to treat patients with *α*1AT deficiency and lung emphysema. *α*1AT is a potent inhibitor of the neutrophil enzyme elastase. An imbalance between *α*1AT and elastase increases the risk of emphysema [77]. In a mouse model of silica-induced inflammation, influx of granulocytes in the lungs was significant inhibited by *α*1AT. Interestingly *α*1AT inhibited activation of the proinflammatory transcription factor NF*κ*B in lungs [78]. An *in vitro *study investigated the effect of *α*1AT on the TLR4- mediated activation of human primary monocytes. Only long-term (18 hours) cell exposure to a1AT inhibited LPS-induced upregulation of the proinflammatory mediators TNF-*α*, IL-1*β*, and IL-8. In contrast, short-term exposure to *α*1AT/LPS increased the expression of TNF-*α*, IL-1*β*, and IL-8. Furthermore, long-term exposure of monocytes to *α*1AT/LPS decreased the surface expression of CD14 and TLR4 [79]. Recently, a study performed by Toldo et al. demonstrated a beneficial effect of *α*1AT in a mouse model of myocardial infarction. Infarct size was significant reduced in the group treated with *α*1AT compared with the control treated with albumin. In addition, *α*1AT treatment significantly reduced cytokine/chemokine tissue levels of IL-6, IL-10, IL-17, TNF-*α*, and MCP-1 [80]. Further investigations are necessary to determine the molecular mechanism of *α*1AT mediated inhibition of TLR-induced activation of cells. Interestingly, it has been speculated by Johnson et al. that elastase released by activated neutrophils might be a major *in vivo* mechanism by which HS is cleaved from the surface of vascular EC and subsequently induces inflammation via TLR4 [81]. 

#### 3.3.6. Reconstituted High-Density Lipoprotein (rHDL)

A beneficial effect of treatment with reconstituted High-Density Lipoprotein (rHDL), containing apolipoprotein A-I and phosphatidylcholine (PC), has been described in multiple diseases including arteriosclerosis, MI, stroke, and endotoxemia. rHDL has been reported to inhibit upregulation of inflammatory adhesion molecules like ICAM-1 (CD54), VCAM-1 (CD106) and E-selectin (CD62E), on EC [82] as well as reduce thrombin-induced tissue-factor (TF) expression [83]. Furthermore, a recent study in humans has shown that rHDL reduces plasma levels of TNF-*α* and expression of CD11b on monocytes [84]. Protection against cardiac I/R injury has been demonstrated in *ex vivo* perfused rat hearts in association with a reduced cardiac content of TNF-*α* and enhanced secretion of prostaglandin [85]. *In vivo*, rHDL has been shown to prevent left ventricle remodeling and improve heart function after MI. Nitric oxide levels in plasma were significantly reduced in the rHDL-treated group, whereas no decrease in IL-6 levels was observed [86]. An effect of rHDL on TLR4- mediated inflammation has been demonstrated in gram-negative sepsis via binding and neutralizing LPS and reduction of CD14 expression on monocytes [87]. Overall, rHDL attenuates the proinflammatory effects of many mediators of innate immunity.

#### 3.3.7. Statins

HMG-CoA reductase inhibitors, namely, statins, have been reported to have broad anti-inflammatory biological properties. In a single center study of HTx patients, statins are even thought to have immunosuppressive properties. Lipid lowering therapy was strongly associated with an improvement in one-year survival of the patients whereas no difference was reported regarding graft rejection [88]. In addition, statins have been demonstrated to inhibit the upregulation of CD86 as well as MHC class II on MoDC and as consequence, allogeneic T cell activation and proliferation was prevented. 

Interestingly, pretreatment of primary human monocytes with atorvastatin and simvastatin significantly inhibited the secretion of IL-6, IL-12, and TNF-*α*. In addition, LPS-induced upregulation of the costimulatory molecule CD80 was significantly inhibited by both statins. Furthermore, atorvastatin as well as simvastatin induced a decrease in TLR4 expression at the protein as well as the mRNA level in human monocytes [89]. In addition, statins have been demonstrated to inhibit the upregulation of CD86 as well as MHC class II on MoDC and as a consequence, allogeneic T cell activation and proliferation were prevented [90].

## 4. Discussion

Accumulating evidence indicates that TLR may play important roles in the pathogenesis of atherosclerosis, microvascular complication of diabetes, viral myocarditis, dilated cardiomyopathy, cardiac allograft rejection, and sepsis-induced left ventricular dysfunction. Moreover, heart failure of diverse etiology is also now recognized to have an important immune component, with TLR signaling influencing the process of cardiac remodeling and prognosis. In cardiac I/R injury, studies with TLR2 and TLR4 deficient mice have suggested a crucial involvement of these receptors in the early process of inflammation. In addition, blockade with TLR2 or TLR4 antagonists has been shown to have a beneficial effect in MI [42, 43]. Several danger molecules or DAMP identified *in vitro* are suggested to be the ligands of TLR and therefore the initiators of sterile inflammation, but overall, there is still no direct evidence *in vivo* that these molecules are the drivers of the inflammatory process. Cardiac I/R is a multifactorial injury resulting in the activation of several proinflammatory pathways such as the complement or coagulation system (Figure 2). Inhibition of complement has been demonstrated to be cardioprotective in I/R injury but had no effect on patients treated with fibrinolysis for acute MI [91]. There are several compounds described, most of them already routinely used in the clinic to treat patients, for example, C1-INH or IVIg, which inhibit various proinflammatory pathways including TLR. Antagonism of these pattern recognition receptors might contribute to the observed therapeutic effect of these substances shown in various animal models of cardiac I/R injury. Most of the “nonselective” TLR antagonists summarized in Table 1 seem to mainly target TLR4, but in almost all studies, only LPS was used as activating agent. Whether these compounds might also inhibit TLR signaling induced by other TLR ligands, exogenous as well as endogenous, remains to be investigated. Despite the large amount of evidence, based largely on animal studies, that TLRs are involved in a wide range of pathological cardiovascular processes, clinical translation of therapeutic targeting of TLR in CVD in man is still in the early stages. Two TLR4 antagonists, E5564 (Eritoran) by Eisai, Inc., and TAK-242 by Takeda Pharmaceutical Company, have been used in phase III clinical trials for the management of severe sepsis where the drugs ware largely well tolerated. For cardiovascular diseases, Eritoran also showed some preclinical benefits and attenuated myocardial I/R injury by inhibiting TLR4. Currently, there are no additionally published clinical trial data looking at TLR antagonists as a therapeutic for CVD (Table 2).

This slow progress may have several causes. In most cases, it is unclear as to how and to what extent various TLR receptors and signaling pathways are altered in different CVDs. In virtually all cases, how the complete family of TLR is altered in different disease states is unknown. This may have various consequences particularly if activation of specific TLR has protective effects. Effective therapeutic intervention may require antagonist blockade of detrimental receptors and simultaneous agonist treatment of beneficial TLR receptor responses. Another major drawback may be the limitations of currently used animal models to mimic the clinical entity being targeted. In most cases, CVD occurs against a background of one or more other pathological processes such as hyperlipidemia, atherosclerosis, and hyperglycemia each of which may differentially modify the TLR receptor and signaling profile in any given tissue or cell type. Rarely are experimental therapeutic profiles performed in atherosclerotic prone diabetic mice when evaluating, for example, peripheral arterial disease. Until the profiles of TLR changes are fully understood, it is unlikely that monotherapy targeting a single TLR entity will prove to be effective in treating CVD.

Thus, more experiments are warranted to study the detailed profile of TLR changes in CVD and the differential or additive effects of one or more risk factors that are known to influence disease progression or outcome.

## Figures and Tables

**Table 1:** Non selective-TLR antagonists in cardiovascular disease.

Compound	*In vitro* (TLR inhibition)		*In vivo* (cardiac I/R injury, HTx)
	TLR/cells	TLR agonists	
LMW-DXS	TLR2 and TLR4/human MoDC [33] TLR2/human NK cells [50]	TLR2: LTA, Pam3CSK4 TLR4: LPS, HS	MI/pig [48] HTx/rat [49] Xeno HTx/hamster-to-rat [92]
IVIg	TLR4/human MoDC [64] TLR9/human B cells [66]	TLR4: LPS TLR9: CpG Oligos	HTx/human [63]
C1-INH	TLR4/murine macrophage cell line RAW264.7 [70]	TLR4: LPS	MI/pig [68] MI/cat [69] HTx/human [63]
ATIII	TLR4/human monocytic cell line THP1 [75]	TLR4: LPS	HTx/mice [76]
*α*1AT	TLR4/human monocytes [79]	TLR4: LPS	MI/mice [80]
rHDL	TLR4/human monocytes [87]	TLR4: LPS	MI/rat [86]
Statins	TLR4/human monocytes [89] TLR4/human MoDC [90]	TLR4: LPS	MI/human [93] HTx/human [88]

HS: heparan sulfate; HTx: heart transplantation; MI: myocardial infarction; MoDC: monocyte-derived dendritic cells: LTA: lipoteichoic acid; LPS: lipopolysaccharide.

**Table 2:** Selective-TLR antagonists in cardiovascular disease.

Compound	Target	Indication	Drug class	Clinical phase	Company
OPN-305	TLR2	Inflammation, autoimmunity, I/R injury	Antibody	Preclinical	Opsona Therapeutics
Eritoran	TLR4	Sepsis I/R injury (preclinical)	Synthetic lipopolysacharide	Phase III	Eisai Pharmaceuticals
TAK-242	TLR4	Sepsis, inflammation	Small-molecule inhibitor	Discontinued in phase III	Takeda
NI-0101	TLR4	Inflammation, autoimmunity, CVD, I/R injury, and so forth	Antibody	Preclinical	Novimmune
IMO-3100	TLR7 and TLR9	SLE, RA, MS, atherosclerosis	DNA-based compound	Preclinical	Idera Pharmaceuticals

CVD: cardiovascular disease; MS: multiple sclerosis; RA: rheumatoid arthritis; SLE: systemic lupus erythematosus; I/R: ischaemia reperfusion.

## Abbreviations

CVD: Cardiovascular Disease
DC: Dendritic cell
DAMP: damage-associated-molecular-pattern
HTx: Heart Transplantation
I/R injury: Ischemia/Reperfusion injury
IVIg: Intravenous Immunoglobulins
LMW-DXS: Low molecular weight dextran sulfate
MI: Myocardial infarction
MoDC: monocyte-derived dendritic cells
TLR: Toll-like receptors.

## References

[1] Danese S, Dejana E, Fiocchi C (2007). Immune regulation by microvascular endothelial cells: directing innate and adaptive immunity, coagulation, and inflammation. *Journal of Immunology*.

[2] Lemaitre B, Nicolas E, Michaut L, Reichhart JM, Hoffmann JA (1996). The dorsoventral regulatory gene cassette spatzle/Toll/Cactus controls the potent antifungal response in Drosophila adults. *Cell*.

[3] Medzhitov R, Preston-Hurlburt P, Janeway CA (1997). A human homologue of the Drosophila toll protein signals activation of adaptive immunity. *Nature*.

[4] Beutler B (2004). Inferences, questions and possibilities in Toll-like receptor signalling. *Nature*.

[5] Alegre ML, Leemans J, Le Moine A (2008). The multiple facets of toll-like receptors in transplantation biology. *Transplantation*.

[6] Akira S, Takeda K (2004). Toll-like receptor signalling. *Nature Reviews Immunology*.

[7] O'Neill LAJ (2006). How Toll-like receptors signal: what we know and what we don't know. *Current Opinion in Immunology*.

[8] Kaisho T, Takeuchi O, Kawai T, Hoshino K, Akira S (2001). Endotoxin-induced maturation of MyD88-deficient dendritic cells. *Journal of Immunology*.

[9] Kawai T, Takeuchi O, Fujita T (2001). Lipopolysaccharide stimulates the MyaD88-independent pathway and results in activation of IFN-regulatory factor 3 and the expression of a subset of lipopolysaccharide-inducible genes. *Journal of Immunology*.

[10] Taylor KR, Yamasaki K, Radek KA (2007). Recognition of hyaluronan released in sterile injury involves a unique receptor complex dependent on toll-like receptor 4, CD44, and MD-2. *Journal of Biological Chemistry*.

[11] Matzinger P (1994). Tolerance, danger, and the extended family. *Annual Review of Immunology*.

[12] Gallucci S, Lolkema M, Matzinger P (1999). Natural adjuvants: endogenous activators of dendritic cells. *Nature Medicine*.

[13] Lin E, Freedman JE, Beaulieu LM (2009). Innate immunity and toll-like receptor antagonists: a potential role in the treatment of cardiovascular diseases. *Cardiovascular Therapeutics*.

[14] Kono H, Rock KL (2008). How dying cells alert the immune system to danger. *Nature Reviews Immunology*.

[15] Chen GY, Nuñez G (2010). Sterile inflammation: sensing and reacting to damage. *Nature Reviews Immunology*.

[16] Higashimori M, Tatro JB, Moore KJ, Mendelsohn ME, Galper JB, Beasley D (2011). Role of toll-like receptor 4 in intimal foam cell accumulation in apolipoprotein E-deficient mice. *Arteriosclerosis, Thrombosis, and Vascular Biology*.

[17] Gu J-Q, Wang D-F, Yan X-G (2010). A Toll-like receptor 9-mediated pathway stimulates perilipin 3 (TIP47) expression and induces lipid accumulation in macrophages. *American Journal of Physiology*.

[18] LeBouder E, Rey-Nores JE, Rushmere NK (2003). Soluble forms of Toll-like receptor (TLR)2 capable of modulating TLR2 signaling are present in human plasma and breast milk. *Journal of Immunology*.

[19] Ueland T, Espevik T, Kjekshus J (2006). Mannose binding lectin and soluble Toll-like receptor 2 in heart failure following acute myocardial infarction. *Journal of Cardiac Failure*.

[20] Cole JE, Navin TJ, Cross AJ (2011). Unexpected protective role for Toll-like receptor 3 in the arterial wall. *Proceedings of the National Academy of Sciences of the United States of America*.

[21] Shi H, Kokoeva MV, Inouye K, Tzameli I, Yin H, Flier JS (2006). TLR4 links innate immunity and fatty acid-induced insulin resistance. *Journal of Clinical Investigation*.

[22] Tarkowski A, Bjersing J, Shestakov A, Bokarewa MI (2010). Resistin competes with lipopolysaccharide for binding to Toll-like receptor 4. *Journal of Cellular and Molecular Medicine*.

[23] Buraczynska M, Baranowicz-Gaszczyk I, Tarach J, Ksiazek A (2009). Toll-like receptor 4 gene polymorphism and early onset of diabetic retinopathy in patients with type 2 diabetes. *Human Immunology*.

[24] Devaraj S, Jialal I, Yun JM, Bremer A (2011). Demonstration of increased toll-like receptor 2 and toll-like receptor 4 expression in monocytes of type 1 diabetes mellitus patients with microvascular complications. *Metabolism*.

[25] Hodgkinson CP, Laxton RC, Patel K, Ye S (2008). Advanced glycation end-product of low density lipoprotein activates the toll-like 4 receptor pathway implications for diabetic atherosclerosis. *Arteriosclerosis, Thrombosis, and Vascular Biology*.

[26] Kaczorowski DJ, Nakao A, Mollen KP (2007). Toll-like receptor 4 mediates the early inflammatory response after cold ischemia/reperfusion. *Transplantation*.

[27] Favre J, Musette P, Douin-Echinard V (2007). Toll-like receptors 2-deficient mice are protected against postischemic coronary endothelial dysfunction. *Arteriosclerosis, Thrombosis, and Vascular Biology*.

[28] Zhao P, Wang J, He L (2009). Deficiency in TLR4 signal transduction ameliorates cardiac injury and cardiomyocyte contractile dysfunction during ischemia. *Journal of Cellular and Molecular Medicine*.

[29] Ha T, Hu Y, Liu L (2010). TLR2 ligands induce cardioprotection against ischaemia/reperfusion injury through a PI3K/Akt-dependent mechanism. *Cardiovascular Research*.

[30] Zhang M, Alicot EM, Chiu I (2006). Identification of the target self-antigens in reperfusion injury. *Journal of Experimental Medicine*.

[31] Hawlisch H, Belkaid Y, Baelder R, Hildeman D, Gerard C, Köhl J (2005). C5a negatively regulates toll-like receptor 4-induced immune responses. *Immunity*.

[32] Zhang X, Kimura Y, Fang C (2007). Regulation of Toll-like receptor-mediated inflammatory response by complement in vivo. *Blood*.

[33] Spirig R, Van Kooten C, Obregon C, Nicod L, Daha M, Rieben R (2008). The complement inhibitor low molecular weight dextran sulfate prevents TLR4-induced phenotypic and functional maturation of human dendritic cells. *Journal of Immunology*.

[34] Castellano G, Woltman AM, Nauta AJ (2004). Maturation of dendritic cells abrogates C1q production in vivo and in vitro. *Blood*.

[35] Baruah P, Dumitriu IE, Peri G (2006). The tissue pentraxin PTX3 limits C1q-mediated complement activation and phagocytosis of apoptotic cells by dendritic cells. *Journal of Leukocyte Biology*.

[36] Baruah P, Dumitriu IE, Malik TH (2009). C1q enhances IFN-7 production by antigen-specific T cells via the CD40 costimulatory pathway on dendritic cells. *Blood*.

[37] Spirig R, Djafarzadeh S, Regueira T (2010). Effects of TLR agonists on the hypoxia-regulated transcription factor HIF-1*α* and dendritic cell maturation under normoxic conditions. *PLoS One*.

[38] Jantsch J, Chakravortty D, Turza N (2008). Hypoxia and hypoxia-inducible factor-1 alpha modulate lipopolysaccharide-induced dendritic cell activation and function. *Journal of Immunology*.

[39] Brunner F, Du Toit EF, Opie LH (1992). Endothelin release during ischaemia and reperfusion of isolated perfused rat hearts. *Journal of Molecular and Cellular Cardiology*.

[40] Ehrenreich H, Anderson RW, Fox CH (1990). Endothelins, peptides with potent vasoactive properties, are produced by human macrophages. *Journal of Experimental Medicine*.

[41] Spirig R, Potapova I, Shaw-Boden J, Tsui J, Rieben R, Shaw SG (2009). TLR2 and TLR4 agonists induce production of the vasoactive peptide endothelin-1 by human dendritic cells. *Molecular Immunology*.

[42] Arslan F, Smeets MB, O’Neill LAJ (2010). Myocardial ischemia/reperfusion injury is mediated by leukocytic toll-like receptor-2 and reduced by systemic administration of a novel anti-toll-like receptor-2 antibody. *Circulation*.

[43] Shimamoto A, Chong AJ, Yada M (2006). Inhibition of toll-like receptor 4 with eritoran attenuates myocardial ischemia-reperfusion injury. *Circulation*.

[44] Pangburn MK, Atkinson MAL, Meri S (1991). Localization of the heparin-binding site on complement factor H. *Journal of Biological Chemistry*.

[45] Wuillemin WA, Te Velthuis H, Lubbers YTP, De Ruig CP, Eldering E, Hack CE (1997). Potentiation of C1 inhibitor by glycosaminoglycans: dextran sulfate species are effective inhibitors of in vitro complement activation in plasma. *Journal of Immunology*.

[46] Wuillemin WA, Eldering E, Citarella F, De Ruig CP, Cate HT, Erik Hack C (1996). Modulation of contact system proteases by glycosaminoglycans: selective enhancement of the inhibition of factor XIa. *Journal of Biological Chemistry*.

[47] Zeerleder S, Mauron T, Lämmle B, Wuillemin WA (2002). Effect of low-molecular weight dextran sulfate on coagulation and platelet function tests. *Thrombosis Research*.

[48] Banz Y, Hess OM, Robson SC (2005). Locally targeted cytoprotection with dextran sulfate attenuates experimental porcine myocardial ischaemia/reperfusion injury. *European Heart Journal*.

[49] Gajanayake T, Sawitzki B, Matozan K (2008). Dextran sulfate facilitates anti-CD4 mAb-induced long-term rat cardiac allograft survival after prolonged cold ischemia. *American Journal of Transplantation*.

[50] Millard AL, Spirig R, Mueller NJ, Seebach JD, Rieben R (2010). Inhibition of direct and indirect TLR-mediated activation of human NK cells by low molecular weight dextran sulfate. *Molecular Immunology*.

[51] Platt JL, Vercellotti GM, Lindman BJ, Oegema TR, Bach FH, Dalmasso AP (1990). Release of heparan sulfate from endothelial cells. Implications for pathogenesis of hyperacute rejection. *Journal of Experimental Medicine*.

[52] Platt JL, Dalmasso AP, Lindman BJ, Ihrcke NS, Bach FH (1991). The role of C5a and antibody in the release of heparan sulfate from endothelial cells. *European Journal of Immunology*.

[53] Ihrcke NS, Platt JL (1996). Shedding of heparan sulfate proteoglycan by stimulated endothelial cells: evidence for proteolysis of cell-surface molecules. *Journal of Cellular Physiology*.

[54] Ihrcke NS, Parker W, Reissner KJ, Platt JL (1998). Regulation of platelet heparanase during inflammation: role of pH and proteinases. *Journal of Cellular Physiology*.

[55] Rehm M, Bruegger D, Christ F (2007). Shedding of the endothelial glycocalyx in patients undergoing major vascular surgery with global and regional ischemia. *Circulation*.

[56] Johnson GB, Brunn GJ, Kodaira Y, Platt JL (2002). Receptor-mediated monitoring of tissue well-being via detection of soluble heparan sulfate by toll-like receptor 4. *Journal of Immunology*.

[57] Wrenshall LE, Stevens RB, Cerra FB, Platt JL (1999). Modulation of macrophage and B cell function by glycosaminoglycans. *Journal of Leukocyte Biology*.

[58] Wrenshall LE, Cerra FB, Singh RK, Platt JL (1995). Heparan sulfate initiates signals in murine macrophages leading to divergent biologic outcomes. *Journal of Immunology*.

[59] Schmidt P, Magnusson C, Lundgren T, Korsgren O, Nilsson B (2008). Low molecular weight dextran sulfate is well tolerated in humans and increases endogenous expression of islet protective hepatocyte growth factor. *Transplantation*.

[60] Nimmerjahn F, Ravetch JV (2008). Anti-inflammatory actions of intravenous immunoglobulin. *Annual Review of Immunology*.

[61] Durandy A, Kaveri SV, Kuijpers TW (2009). Intravenous immunoglobulins-understanding properties and mechanisms. *Clinical and Experimental Immunology*.

[62] Tha-In T, Metselaar HJ, Bushell AR, Kwekkeboom J, Wood KJ (2010). Intravenous immunoglobulins promote skin allograft acceptance by triggering functional activation of CD4+Foxp3+ T cells. *Transplantation*.

[63] Mohacsi P, Rieben R, Sigurdsson G (2001). Successful management of a B-type cardiac allograft into an O-type man with 3 1/2-year clinical follow-up. *Transplantation*.

[64] Bayry J, Lacroix-Desmazes S, Carbonneil C (2003). Inhibition of maturation and function of dendritic cells by intravenous immunoglobulin. *Blood*.

[65] Ballow M, Allen C (2011). Intravenous immunoglobulin modulates the maturation of TLR 4-primed peripheral blood monocytes. *Clinical Immunology*.

[66] Kessel A, Peri R, Haj T (2011). IVIg attenuates TLR-9 activation in B cells from SLE patients. *Journal of Clinical Immunology*.

[67] Davis AE, Mejia P, Lu F (2008). Biological activities of C1 inhibitor. *Molecular Immunology*.

[68] Horstick G, Berg O, Heimann A (2001). Application of C1-esterase inhibitor during reperfusion of ischemic myocardium: dose-related beneficial versus detrimental effects. *Circulation*.

[69] Buerke M, Murohara T, Lefer AM (1995). Cardioprotective effects of a C1 esterase inhibitor in myocardial ischemia and reperfusion. *Circulation*.

[70] Liu D, Cai S, Gu X, Scafidi J, Wu X, Davis AE (2003). C1 inhibitor prevents endotoxin shock via a direct interaction with lipopolysaccharide. *Journal of Immunology*.

[71] Liu D, Gu X, Scafidi J, Davis AE (2004). N-linked glycosylation is required for C1 inhibitor-mediated protection from endotoxin shock in mice. *Infection and Immunity*.

[72] Bergamaschini L, Gobbo G, Gatti S (2001). Endothelial targeting with C1-inhibitor reduces complement activation in vitro and during ex vivo reperfusion of pig liver. *Clinical and Experimental Immunology*.

[73] Patnaik MM, Moll S (2008). Inherited antithrombin deficiency: a review. *Haemophilia*.

[74] Ostrovsky L, Woodman RC, Payne D, Teoh D, Kubes P (1997). Antithrombin III prevents and rapidly reverses leukocyte recruitment in ischemia/reperfusion. *Circulation*.

[75] Mansell A, Reinicke A, Worrall DM, O’Neill LAJ (2001). The serine protease inhibitor antithrombin III inhibits LPS-mediated NF-*κ*B activation by TLR-4. *FEBS Letters*.

[76] Aramaki O, Takayama T, Yokoyama T (2003). High dose of antithrombin III induces indefinite survival of fully allogeneic cardiac grafts and generates regulatory cells. *Transplantation*.

[77] Silverman EK, Sandhaus RA (2009). Alpha_1_-antitrypsin deficiency. *New England Journal of Medicine*.

[78] Churg A, Dai J, Zay K (2001). Alpha-1-antitrypsin and a broad spectrum metalloprotease inhibitor, RS113456, have similar acute anti-inflammatory effects. *Laboratory Investigation*.

[79] Nita IM, Serapinas D, Janciauskiene SM (2007). *α*1-Antitrypsin regulates CD14 expression and soluble CD14 levels in human monocytes in vitro. *International Journal of Biochemistry and Cell Biology*.

[80] Toldo S, Seropian IM, Mezzaroma E (2011). Alpha-1 antitrypsin inhibits caspase-1 and protects from acute myocardial ischemia-reperfusion injury. *Journal of Molecular and Cellular Cardiology*.

[81] Johnson GB, Brunn GJ, Platt JL (2004). Cutting edge: an endogenous pathway to systemic inflammatory response syndrome (SIRS)-like reactions through Toll-like receptor 4. *Journal of Immunology*.

[82] Wadham C, Albanese N, Roberts J (2004). High-density lipoproteins neutralize C-reactive protein proinflammatory activity. *Circulation*.

[83] Viswambharan H, Ming XF, Zhu S (2004). Reconstituted high-density lipoprotein inhibits thrombin-induced endothelial tissue factor expression through inhibition of RhoA and stimulation of phosphatidylinositol 3-kinase but not Akt/endothelial nitric oxide synthase. *Circulation Research*.

[84] Shaw JA, Bobik A, Murphy A (2008). Infusion of reconstituted high-density lipoprotein leads to acute changes in human atherosclerotic plaque. *Circulation Research*.

[85] Calabresi L, Rossoni G, Gomaraschi M, Sisto F, Berti F, Franceschini G (2003). High-density lipoproteins protect isolated rat hearts from ischemia-reperfusion injury by reducing cardiac tumor necrosis factor-*α* content and enhancing prostaglandin release. *Circulation Research*.

[86] Kiya Y, Miura SI, Imaizumi S (2009). Reconstituted high-density lipoprotein attenuates postinfarction left ventricular remodeling in rats. *Atherosclerosis*.

[87] Pajkrt D, Doran JE, Koster F (1996). Antiinflammatory effects of reconstituted high-density lipoprotein during human endotoxemia. *Journal of Experimental Medicine*.

[88] Kobashigawa JA (2004). Statins in solid organ transplantation: is there an immunosuppressive effect?. *American Journal of Transplantation*.

[89] Methe H, Kim J-O, Kofler S, Nabauer M, Weis M (2005). Statins decrease Toll-like receptor 4 expression and downstream signaling in human CD14^+^ monocytes. *Arteriosclerosis, Thrombosis, and Vascular Biology*.

[90] Yilmaz A, Reiss C, Weng A (2006). Differential effects of statins on relevant functions of human monocyte-derived dendritic cells. *Journal of Leukocyte Biology*.

[91] Mahaffey KW, Granger CB, Nicolau JC (2003). Effect of pexelizumab, an anti-C5 complement antibody, as adjunctive therapy to fibrinolysis in acute myocardial infarction: the COMPlement inhibition in myocardial infarction treated with thromboLYtics (COMPLY) trial. *Circulation*.

[92] Laumonier T, Mohacsi PJ, Matozan KM (2004). Endothelial cell protection by dextran sulfate: a novel strategy to prevent acute vascular rejection in xenotransplantation. *American Journal of Transplantation*.

[93] Stenestrand U, Wallentin L (2001). Early statin treatment following acute myocardial infarction and 1-year survival. *Journal of the American Medical Association*.

